# Microorganisms resistant to conventional antimicrobials in acute exacerbations of chronic obstructive pulmonary disease

**DOI:** 10.1186/s12931-018-0820-1

**Published:** 2018-06-15

**Authors:** Cristina Estirado, Adrian Ceccato, Monica Guerrero, Arturo Huerta, Catia Cilloniz, Olivia Vilaró, Albert Gabarrús, Joaquim Gea, Ernesto Crisafulli, Nestor Soler, Antoni Torres

**Affiliations:** 1 Pulmonology Department, Hospital del Mar-IMIM. CEXS, Universitat Pompeu Fabra, CIBERES, ISCiii, Barcelona, Spain; 2 Pneumology Department, Respiratory Institute (ICR), Hospital Clinic of Barcelona - Institut d’Investigacions Biomèdiques August Pi i Sunyer (IDIBAPS), University of Barcelona (UB), SGR 911- Ciber de Enfermedades Respiratorias (CIBERES), ICREA Academia, Barcelona, Spain; 3 Respiratory and Lung Function Unit, Department of Medicine and Surgery, University of Parma, Parma, Italy; 4 Department of Pneumology, Hospital Clinic of Barcelona, Villarroel 140, 08036 Barcelona, Spain

**Keywords:** COPD, Exacerbation, Resistance to antimicrobials

## Abstract

**Background:**

Antimicrobial treatment for acute exacerbations of chronic obstructive pulmonary disease (AECOPD) remains controversial. In some cases AECOPD are caused by microorganisms that are resistant to treatments recommended by guidelines. Our aims were: 1) identify the risk factors associated with infection by microorganisms resistant to conventional treatment (MRCT), 2) Compare the clinical characteristics and outcomes of patients with AECOPD resulting from MRCT against those with AECOPD from other causes.

**Methods:**

We prospective analysed a cohort of patients admitted with severe AECOPD (2009 to 2015) who were assigned to three groups: patients with MRCT (those patients with germs resistant to antibiotics recommended in guidelines), patients with microorganisms sensitive to conventional antimicrobial treatment (MSCT), and patients with negative microbiology results who had not previously received antibiotics. Multinomial logistic regression analyses were used to examine the associations between microbial aetiology groups and risk factors. The association between LOS and risk factors was also tested in simple and multiple analyses, and similar inclusion criteria were applied for the linear regression analysis.

**Results:**

Of the 451 patients admitted, 195 patients (43%) were included. Respiratory cultures were positive in 86(44%) and negative in 109(56%). MRCT were isolated in 34 cases (40%) and MSCT in 52 (60%). Patients with MRCT had more AECOPD in the previous year, received more antibiotic treatment in the previous three months, had more severe disease, higher dyspnoea and a positive respiratory culture in the previous year (mainly for *Pseudomonas aeruginosa*). The following conditions were independent factors for MRCT isolation: non-current smoker (odds ratio [OR] 4.19 [95% confidence interval [CI] 1.29–13.67], *p* = 0.017), ≥ 2 AECOPD or ≥ 1 admission for AECOPD in the previous year (OR 4.13 [95% CI 1.52–11.17], *p* = 0.005), C-reactive protein < 5 mg/dL; (OR 3.58 [95% CI 1.41–9.07], *p* = 0.007). Mortality rates were comparable at 30-days, one year and 3 years; however, patients in the MRCT group had longer hospital stays.

**Conclusion:**

In conclusion, there are risk factors for resistant germs in AECOPD; however, the presence of these germs does not increase mortality. Patients with isolation of MRCT had longer length of stay.

**Electronic supplementary material:**

The online version of this article (10.1186/s12931-018-0820-1) contains supplementary material, which is available to authorized users.

## Background

Acute exacerbations of chronic obstructive pulmonary disease (AECOPD) negatively affect hospitalisation, readmission, disease progression and mortality rates in patients with COPD [1]. Severe AECOPD are mainly triggered by bacterial infection, viral infection or environmental agents, with the most common causes of bacterial infection being *Streptococcus pneumoniae, Haemophilus influenzae* and *Moraxella catarrhalis* [2–4]. Thus, current recommendations for antimicrobial treatment are aminopenicillin with or without clavulanic acid, a macrolide or a tetracycline [5, 6]. AECOPD are infrequently caused by microorganisms—such as *Pseudomonas aeruginosa*, methicillin-resistant *Staphylococcus aureus* (MRSA), *Stenotrophomonas maltophilia* or enterobacteria—that are resistant to these treatments. Guidelines and previous studies of severe AECOPD suggest that these patients have increased frequencies of exacerbations, previous antibiotic use, previous hospital admissions and more severe airflow limitations [2, 5, 7, 8].

At least 30% of COPD patients are colonised by a potential pathogen when in a stable phase of their disease; however only 0.5% are colonised by *Enterobacteriaceae*, *P. aeruginosa* or *S. maltophilia* [9]. Also, AECOPD are associated with the overgrowth of potential pathogens and with the occurrence of *P. aeruginosa* in the lower airway [10]. Knowing the risk factors to microorganisms resistant to conventional antibiotic treatment (MRCT) in AECOPD could lead to improved prophylaxis and empirical antimicrobial treatment.

We hypothesised that specific factors predict the presence of MRCT. Our primary aim was to identify the risk factors associated with infection by MRCT. Our secondary aim was to compare the clinical characteristics and outcomes of patients with AECOPD resulting from MRCT against those with AECOPD from other causes.

## Methods

### Study design and patients selection

This observational cohort study was performed between January 2009 and December 2015, and included all patients admitted with a diagnosis of AECOPD to the Respiratory Department of the Hospital Clinic, Barcelona, Spain. COPD was defined according to the Global Initiative for Chronic Obstructive Lung Disease (GOLD) guidelines [5], with spirometry performed in a stable disease phase and at least six months prior to hospital admission. Patients with a smoking history of 10 pack-years were considered positive smokers. A worsening of respiratory symptoms compared with the preceding days, and which required a change in home care medication, was used as a clinical definition for AECOPD [5, 11]. Exacerbation severity was based on the respiratory symptoms/signs and the presence of potential indications for hospitalisation [5]. The exclusion criteria were: 1) documented history of asthma or bronchiectasis as the predominant illness and 2) clinical pneumonia or acute heart failure identified at admission.

### Ethics approval and consent to participate

The study protocol was approved by the Hospital Research and Ethics Committee (CEIC 2008/4106) and the study was conducted in accordance with good clinical practice guidelines and the declaration of Helsinki. Written informed consent was obtained from all enrolled patients.

### Microbiological evaluation

Sputum samples were obtained at admission for bacterial culture, before starting antibiotic therapy. Routine antimicrobial susceptibility testing included the disc diffusion method or E-test for *P. aeruginosa*. The results of susceptibility testing were interpreted according to the European Committee on Antimicrobial Susceptibility Testing guidelines [12]. Multidrug-resistant (MDR), extensively drug resistant (XDR) and pan-drug resistant bacteria were categorised according to criteria set out by Magiorakos et al. [13]. The quality of sputum samples was assessed using the Murray and Washington scoring system. Patients with poor quality sputum samples (> 10 epithelial cells or < 25 leucocytes) were excluded from analysis [14]. Patients with mycobacterial, fungal isolation (e.g., Aspergillus or Candida) or *Nocardia spp.* were also excluded.

Patients were classified into 3 groups: 1) patients with the isolation of microorganism sensitive to conventional treatment (MSCT) according to GOLD guidelines (i.e., aminopenicillin with clavulanic acid, a macrolide or a tetracycline); 2) patients with MRCT isolation, (i.e., *P. aeruginosa,* MRSA*, S. maltophilia, Enterobacteriaceae* producer of extended spectrum of beta lactamase and *Acinetobacter baumannii*); and 3) patients with negative microbiology results who did not receive antibiotics in the 7 days previous at admission.

Previous antibiotic treatment was not considered as inclusion/exclusion criteria in MRCT or MSCT groups. Nobody patient used macrolide as chronic treatment, thus it was not considered as variable.

### Clinical measurements and outcomes

Demographic variables, body mass index (BMI), smoking history (former smoker was considered as those patients who quit smoke more than 12 months), presence of co-morbidities measured by Charlson index [15], baseline dyspnoea grade based in modified medical research council (mMRC), COPD severity score measured by a questionnaire (COPDSS) [16] and BODEx index (i.e., BMI, airflow obstruction, dyspnoea and exacerbations) [17], use of long-term oxygen therapy (LTOT) and use of domiciliary medications (i.e., inhaled bronchodilators, such as short-acting β_2_ agonist [SABA], long-acting β_2_ agonist [LABA], anticholinergics or inhaled corticosteroids) were recorded at hospital admission. Characteristics of any exacerbations during the previous year, any previous antibiotic treatment (3 months before admission) and any microorganism isolated in the previous year were also recorded. Vital signs (body temperature, respiratory rate, heart rate and blood pressure) were assessed at admission. Arterial blood gases and laboratory parameters (i.e., leukocytes, haematocrit, haemoglobin, C-reactive protein, glucose and creatinine) were recorded at admission and at day 3.

Variables relating to clinical progression included length of hospital stay (LOS), use of non-invasive mechanical ventilation (NIMV), use of invasive mechanical ventilation (IMV) and intensive care unit (ICU) admission during the initial hospitalisation. Data on prognosis (cumulative number of deaths for all-causes and time to death) were recorded at 30 days, 1 year and 3 years.

### Statistical analysis

We report the number and percentage of patients for categorical variables and the median and interquartile range (IQR) for continuous variables. Categorical variables were compared using the chi-square test, and continuous variables were compared by one-way analysis of variance or the nonparametric Kruskal–Wallis test. Post-hoc pairwise comparisons were carried out via the Bonferroni method to control for the experiment-wise error rate. Survival curves were obtained using the Kaplan–Meier method and compared using the Gehan-Breslow-Wilcoxon test. Patients lost to follow-up were censored in the survival analysis.

Multinomial logistic regression analyses were used to examine the associations between microbial aetiology groups (i.e., MRCT or MSCT relative to unknown aetiology) and risk factors (i.e., baseline characteristics and clinical presentation). Variables were included in the multivariate model when univariate comparisons yielded a level of significance of *p* < 0.05 due the limited number of patients in the MRCT and MSCT groups and in order to exclude bias related to overestimation or underestimation of regression coefficient variance. The final multivariate model was calculated in a stepwise forward selection procedure (p_in_ = 0.05, p_out_ = 0.10). To identify the problem of collinearity, we calculated the r coefficient of 2 variables; that is, if 2 independent variables were highly correlated (r > | ± 0.30|), the variable with the largest variance was excluded from the multivariate analysis [18]. The association between LOS and risk factors was also tested in simple and multiple analyses, and similar inclusion criteria were applied for the linear regression analysis (*p* < 0.05). The odds ratios (ORs) or beta coefficients (βs) and their 95% confidence intervals (CIs) were estimated. The Cox and Snell R^2^ and the Nagelkerke R^2^ were calculated to assess the overall fit of the multinomial logistic regression model and the R^2^ for the linear regression model. The area under the receiver operating characteristic (ROC) curve of the multivariate model to predict MRCT was calculated. Internal validation of the prediction models was conducted using ordinary nonparametric bootstrapping with 1000 bootstrap samples and bias-corrected, accelerated, 95% CIs [19]. The same logistic regression analyses for microbial aetiology groups were also performed but using a multinomial logistic regression model for MRCT with only P. aeruginosa or MSCT relative to negative microbiology.

We investigated the missing data patterns for covariates, assumed missing at random [20], and used multiple imputation [21] to generate 5 datasets to evaluate the prediction performance for the microbial aetiology group. The model for multiple imputation included all covariates of the risk models as well as the microbial aetiology group. For simplicity, in the evaluation of the performance we filled in missing values with the first set of imputed values from the multiple imputation.

The level of significance was set at 0.05 (two-tailed). All analyses were performed with IBM SPSS Statistics 23.0 (Armonk, New York).

## Results

### Patient characteristics

**Fig. 1:**
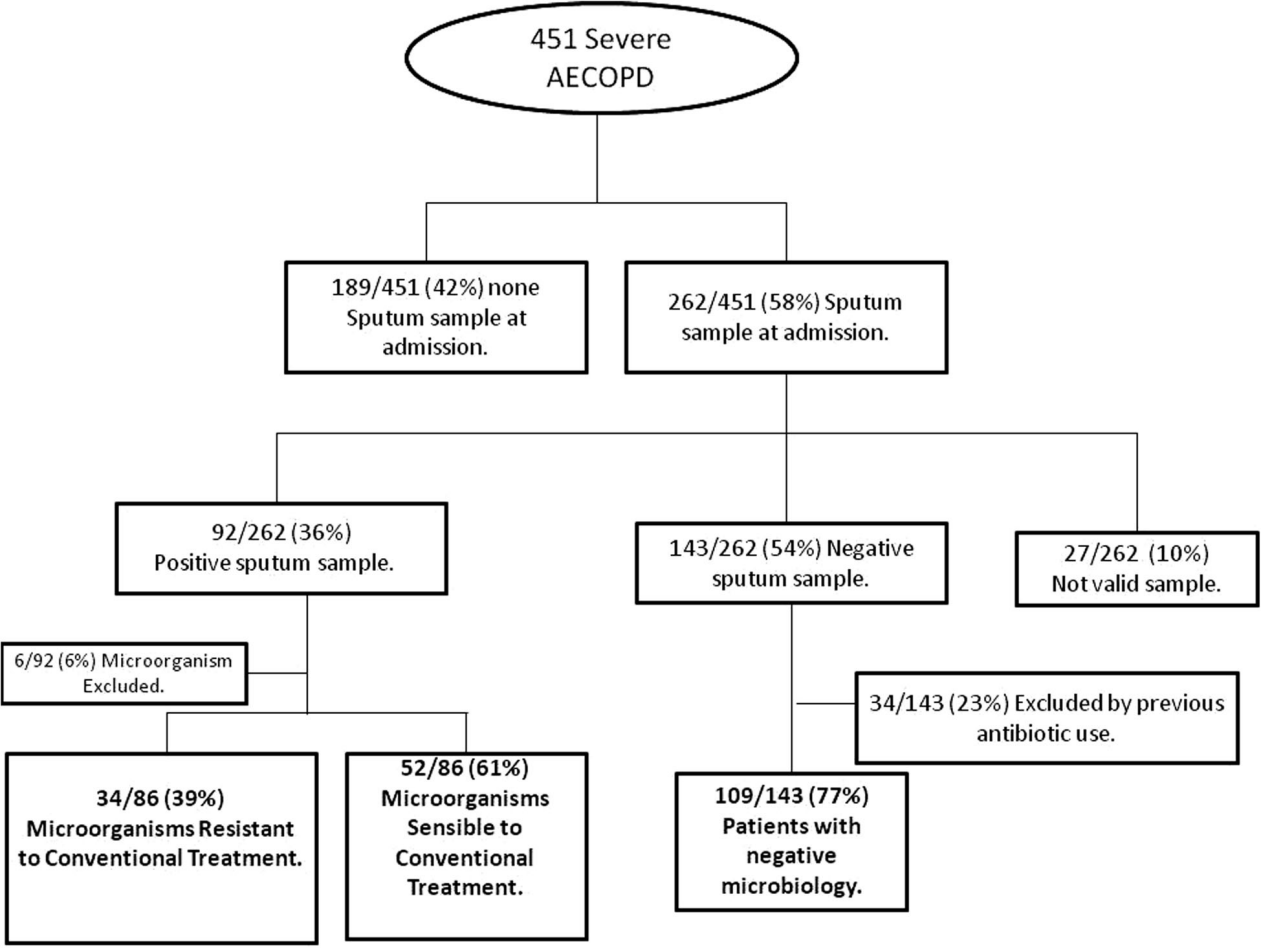
Flowchart

Of the 451 patients admitted with an AECOPD during the observation period, 256 (57%) were excluded. The study population therefore comprised 195 patients (43%), of which 86 (44%) had positive respiratory cultures and 109 (56%) had negative microbiology and no history of previous antibiotic therapy (Fig. 1). AECOPD was associated with MRCT isolation in 34 cases (40%), and other pathogens were isolated in the remaining 52 cases (60%).

Compared with the other groups, patients with MRCT tended to be non-current smokers, have more AECOPD episodes in the previous year, have more hospital admissions in the previous year, receive more antibiotic treatments in the previous 3 months and have more severe disease (higher mMRC dyspnoea grades, higher BODEx indexes and higher COPDSS values) (Table 1). No differences were observed at baseline in purulent sputum, Anthonisen AECOPD classification or pulmonary gas exchange. A higher percentage of patients with MRCT had a positive respiratory culture in the previous year, mainly for *P. aeruginosa.*

**Table 1:** Patient characteristics

Variable	Patients with microorganisms resistant to conventional treatment (*n* = 34)	Patients with microorganisms sensitive to conventional treatment (*n* = 52)	Patients with negative microbiology (*n* = 109)	*P* value
Age, mean (SD), years	73 (10)	71 (10)	71 (11)	0.546
Male sex, n (%)	29 (85)	43 (83)	91 (83)	0.950
BMI, mean (SD), Kg/m^2^	28 (5)	27 (5)	27 (5)	0.756
Current smoker, n (%)	4 (12)^b,c^	21 (40)^a^	43 (39)^a^	***0.008***
Packs/year, median (IQR)	33 (30; 90)	50 (40; 60)	60 (50; 95)	***0.043***
AECOPD in the previous year, n (%)	25 (74)^c^	25 (48)	41 (38)^a^	***0.001***
≥2 AECOPD in the previous year, n (%)	16 (47)^c^	16 (31)	19 (18)^a^	***0.003***
Admissions by AECOPD in the previous year, n (%)	20 (59)^c^	17 (33)	28 (26)^a^	***0.002***
≥ 2 AECOPD or ≥ 1 admission for AECOPD in the previous year, n (%)	24 (71)^b,c^	21 (40)	32 (30)^b^	***< 0.001***
Prior antibiotic treatment (last 3 months), n (%)	26 (79)^b,c^	22 (43)^a,c^	22 (21)^a,b^	***< 0.001***
Prior antibiotic treatment, n (%)	9 (27)	14 (27)	0 (0)	***< 0.001***
Inhaled corticosteroids use, n (%)	17 (50)	19 (41)	35 (38)	0.480
Bronchiectasis, n (%)	13 (48)	10 (28)	29 (38)	0.251
Long-term oxygen therapy, n (%)	18 (53)^c^	15 (29)	31 (28)^a^	***0.023***
Charlson index, median (IQR)	2 (1; 3)	2 (1; 3)	2 (1; 3)	0.459
BODEx index, median (IQR)	3 (0; 6)^c^	0 (0; 4.5)	0 (0; 0)^a^	***0.001***
mMRC Dyspnoea, median (IQR)	3 (2; 3)^b,c^	2 (1; 3)^a^	2 (1; 3)^a^	***< 0.001***
COPDSS, median (IQR)	19 (14; 21)^b,c^	15 (9; 19)^a^	13 (8; 18)^a^	***< 0.001***
FEV_1_, median (IQR), % ref	33 (27; 41)	45 (31; 55)	39 (28; 57)	0.073
FEV_1_ < 35% ref., n (%)	17 (55)	13 (28)	39 (41)	0.064
Positive sputum cultures in the previous year, n (%)	18 (53)^b,c^	12 (23)^a^	11 (10)^a^	***< 0.001***
Pseudomonas aeruginosa in the previous year, n (%)^d^	8 (44)	1 (8)	1 (9)	***0.048***
Respiratory rate, mean (SD)	22 (20; 26)	22 (20; 28)	24 (20; 26)	0.423
Anthonisen classification, n (%)				0.793
Type I	17 (52)	20 (40)	42 (40)	
Type II	9 (27)	19 (38)	38 (36)	
Type III	7 (21)	11 (22)	25 (24)	
Purulent sputum, n (%)	18 (55)	18 (36)	41 (39)	0.203
Haemoglobin, median (IQR), gr/L	134 (120; 146)	142 (127; 151)	139 (124; 153)	0.159
pH, median (IQR)	7.40 (7.36; 7.45)	7.39 (7.34; 7.43)	7.39 (7.35; 7.43)	0.907
PaCO_2_, median (IQR), mmHg	49 (42; 61)	44 (38; 58)	45 (38; 58)	0.340
PaO_2_/FiO_2_, median (IQR), mmHg	257 (230; 321)	248 (207; 293)	267 (232; 311)	0.490
C-reactive protein, median (IQR), mg/dL	2.5 (1.5; 5.4)	5.4 (1.5; 17.4)	4.9 (1.6; 12.8)	0.113

*Abbreviations*: *AECOPD* acute exacerbation of chronic obstructive pulmonary disease, *BMI* body mass index, *BODEx* body mass index, airflow obstruction, dyspnoea and exacerbations, *COPDSS* chronic obstructive pulmonary disease severity score, *FEV*_*1*_ forced expiratory volume in the 1st second, *IQR* interquartile range, *mMRC* modified medical research council, *SD* standard deviation

Data are shown as number and percentage of patients, mean (SD), or median (1st quartile; 3rd quartile)

Percentages are calculated on non-missing data

^a^*P* < 0.05 vs. Patients with microorganisms resistant to conventional treatment

^b^*p* < 0.05 vs. Patients with microorganisms sensitive to conventional treatment

^c^*p* < 0.05 vs. Patients with negative microbiology

^d^Percentages calculated for patients with positive sputum cultures in the previous year

Bold Italic entries indicate statistical significance

### Microbiological findings

**Fig. 2:**
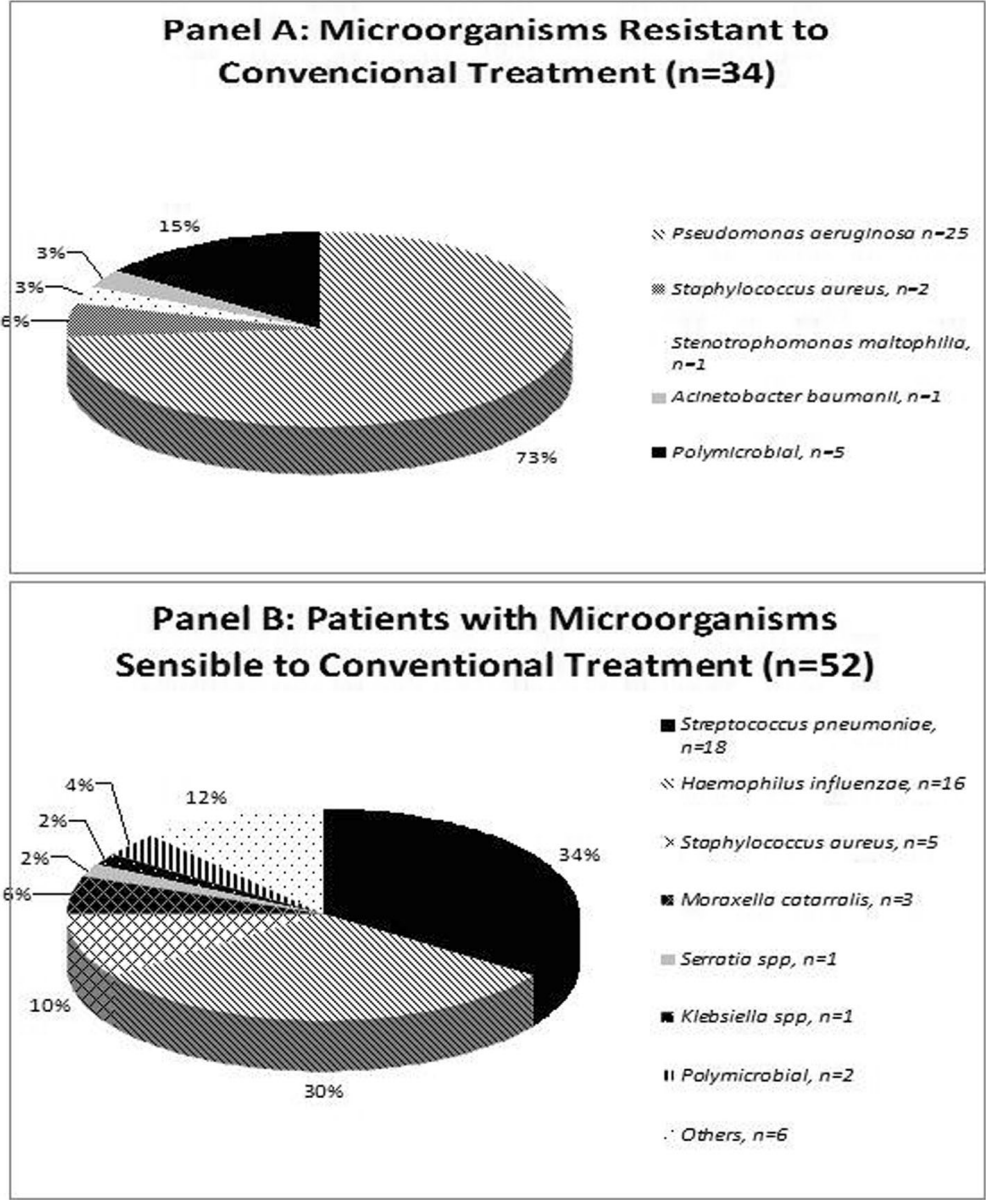
Microbial aetiology. Panel **a** Microorganisms Resistant to Conventional Treatment (*n* = 34). Panel **b** Patients with Microorganisms Sensitive to Conventional Treatment (*n* = 52). Polymicrobial Isolation includes: *3 Pseudomonas aeruginosa, 2 Stenotrophomonas maltophilia, 1 Staphylococcus aureus, 3 Streptococcus pneumoniae, 1 other.* Others isolation includ*e:* 3 Corynebacterium spp., 2 Pasteurella spp., 1 Capnocytophaga spp.

In the group of patients with MRCT, the most frequent pathogen was *P. aeruginosa* (25 patients [74%]). Two patients had methicillin-resistant *S. aureus*, one patient *S. maltophilia*, another had *A. baumannii* and 5 had polymicrobial aetiology (Fig. 2 and Additional file 1: Table S1). In total, 9 patients (50%) with previous *P. aeruginosa* isolation had received effective treatment. Among the patients with MSCT, *S. pneumoniae* and *H. influenzae* were the most common pathogens (35 and 31%, respectively), but 2 patients had *Enterobacteriaceae* (*Klebsiella spp.* and *Serratia spp.*) that were sensitive to aminopenicillins (Fig. 2). *P. aeruginosa* isolates were categorised as MDR in 10 cases (40%) and XDR in 3 cases (12%).

### Antibiotic treatment

Data on antibiotic treatment were available for 163 patients (88%). The most frequent regimen was fluoroquinolone monotherapy (*n* = 82; 50%), penicillin monotherapy (*n* = 27; 17%) and antibiotic combination therapy (*n* = 44; 27%). The group with MRCT received more combination therapy (*p* = 0.003) and less fluoroquinolone monotherapy (*p* = 0.012) compared with the other 2 groups. The most frequent used combinations were β-lactam plus macrolide in the MSCT group and fluoroquinolones based combinations in the MRCT group. Empirical antimicrobial treatment was inadequate in 20 cases with positive microbiology (24%), of which 15 (44%) and 5 (10%) were among patients with MRCT and MSCT, respectively (*p* < 0.001).

### Risk factors for MRCT and MSCT

**Fig. 3:**
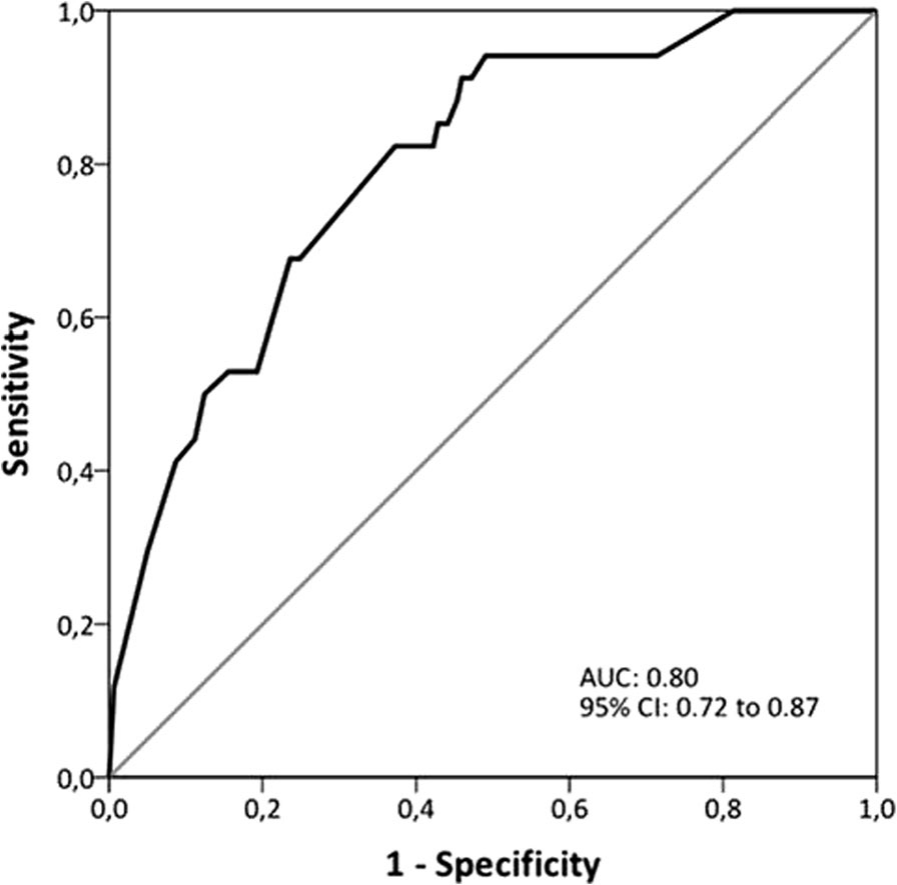
Receiver operating characteristic curve for multinomial logistic regression model to predict MRCT isolation. *Abbreviations*: AUC indicates area under the curve; CI, confidence interval

The following risk factors showed significant associations with the microbial aetiology groups in individual multinomial logistic regression, and were thus used for the initial multivariate model: smoker status, ≥2 AECOPD episode or ≥ 1 AECOPD admission in the previous year, bronchiectasis, LTOT, BODEx index and C-reactive protein (data not shown). The results of the multivariate model are displayed in Table 2. The model shows that the OR for MRCT isolation was significantly increased if the patients was a non-current smoker, had ≥2 AECOPD episodes or ≥ 1 AECOPD admission in the previous year and had a low systemic inflammatory response. The OR for MSCT isolation, however, was strongly decreased with a high BODEx index (4th quartile) (Table 3). The AUC was 0.80 (95% CI, 0.72–0.87) for the model predictive of MRCT isolation (Fig. 3). The data for internal validation of the logistic regression model (using bootstrapping with 1000 samples) are presented in Additional file 1: Table S2. The variables included in the model demonstrated robust results, with small 95% CIs around the original coefficients. When differentiating MRCT with and without *P. aeruginosa,* previous isolation of *P. aeruginosa* was the most important predictor of *P. aeruginosa* isolation (Additional file 1: Table S3, Table S4 and Figure S1).

**Table 2:** Multinomial logistic regression model for microorganisms resistant to conventional treatment or microorganisms sensitive to conventional treatment relative to negative microbiology

Variable	Patients with microorganisms resistant to conventional treatment			Patients with microorganisms sensitive to conventional treatment		
	OR	95% CI	*P* value	OR	95% CI	*P* value
Non-current smoker	4.19	1.29 to 13.67	**0.017**	0.78	0.38 to 1.59	0.49
≥ 2 AECOPD or 1 admission by AECOPD in the previous year	4.13	1.52 to 11.17	**0.005**	1.75	0.76 to 3.99	0.19
BODEx index						
1st quartile: 0–2	1	–	–	1	–	–
2nd quartile: 3–4	2.32	0.67 to 7.98	0.18	0.62	0.21 to 1.88	0.40
3rd quartile: 5–6	1.85	0.58 to 5.90	0.30	1.12	0.44 to 2.88	0.82
4th quartile: 7–9	0.48	0.10 to 2.33	0.37	0.14	0.03 to 0.70	**0.016**
C-reactive protein < 5 mg/dL at admission	3.58	1.41 to 9.07	**0.007**	1.14	0.57 to 2.27	0.72

*Abbreviations: AECOPD* indicates acute exacerbation of chronic obstructive pulmonary disease exacerbation, *BODEx* body mass index, airflow obstruction, dyspnoea and exacerbations, *CI* confidence interval, *OR* odds ratio

Data are shown as estimated ORs (95% CIs) of the explanatory variables observed at admission of patients in the microorganisms resistant to conventional treatment (MRCT) and microorganisms sensitive to conventional treatment (MSCT) groups. The OR is defined as the probability of membership of the groups MRCT or MSCT divided by the probability of membership of the negative microbiology group

The P value is based on the null hypothesis that all ORs relating to an explanatory variable equal unity

Model characteristics: likelihood ratio X^2^ test, *p* = 0.48; R^2^ coefficients = 0.21 (Cox and Snell), 0.24 (Nagelkerke)

**Table 3:** Clinical outcomes

	Patients with microorganisms resistant to conventional treatment (*n* = 34)	Patients with microorganisms sensitive to conventional treatment (*n* = 52)	Patients with negative microbiology (*n* = 109)	*P* value
AECOPD after 30 days of discharge, n (%)	24 (73)	21 (47)	57 (56)	0.070
Number of AECOPD after 30 days of discharge, median (IQR)	1 (0; 3)	0 (0; 2)	1 (0; 1)	0.075
Time to the next AECOPD, median (IQR), days	39 (19; 170)	52 (27; 166)	86 (26; 182)	0.577
Length of stay, median (IQR), days	9 (7; 14)^c^	8 (6; 10.5)	8 (6; 10)^a^	**0.026**
ICU admission, n (%)	4 (12)	6 (12)	12 (11)	0.981
IMV, n (%)	2 (6)	1 (2)	3 (2)	0.564
NIMV, n (%)	6 (18)	10 (19)	16 (15)	0.780
30-day mortality, n (%)	1 (3)	1 (2)	4 (4)	0.834
1-year mortality, n (%)	11 (32)	12 (23)	19 (17)	0.173
3-years mortality, n (%)	16 (59)	19 (56)	40 (43)	0.211

*Abbreviations*: *AECOPD* indicates acute exacerbation of chronic obstructive pulmonary disease exacerbation, *ICU* intensive care unit, *IMV* invasive mechanical ventilation, *IQR* interquartile range, *NIMV* non-invasive mechanical ventilation. Data are shown as number of patients (%), or median (1st quartile; 3rd quartile). Percentages are calculated on non-missing data. ^a^*P* < 0.05 vs. patients with microorganisms resistant to conventional treatment. ^b^*p* < 0.05 vs. patients with microorganisms sensitive to conventional treatment. ^c^*p* < 0.05 vs. patients with negative microbiology

### Outcomes

**Fig. 4:**
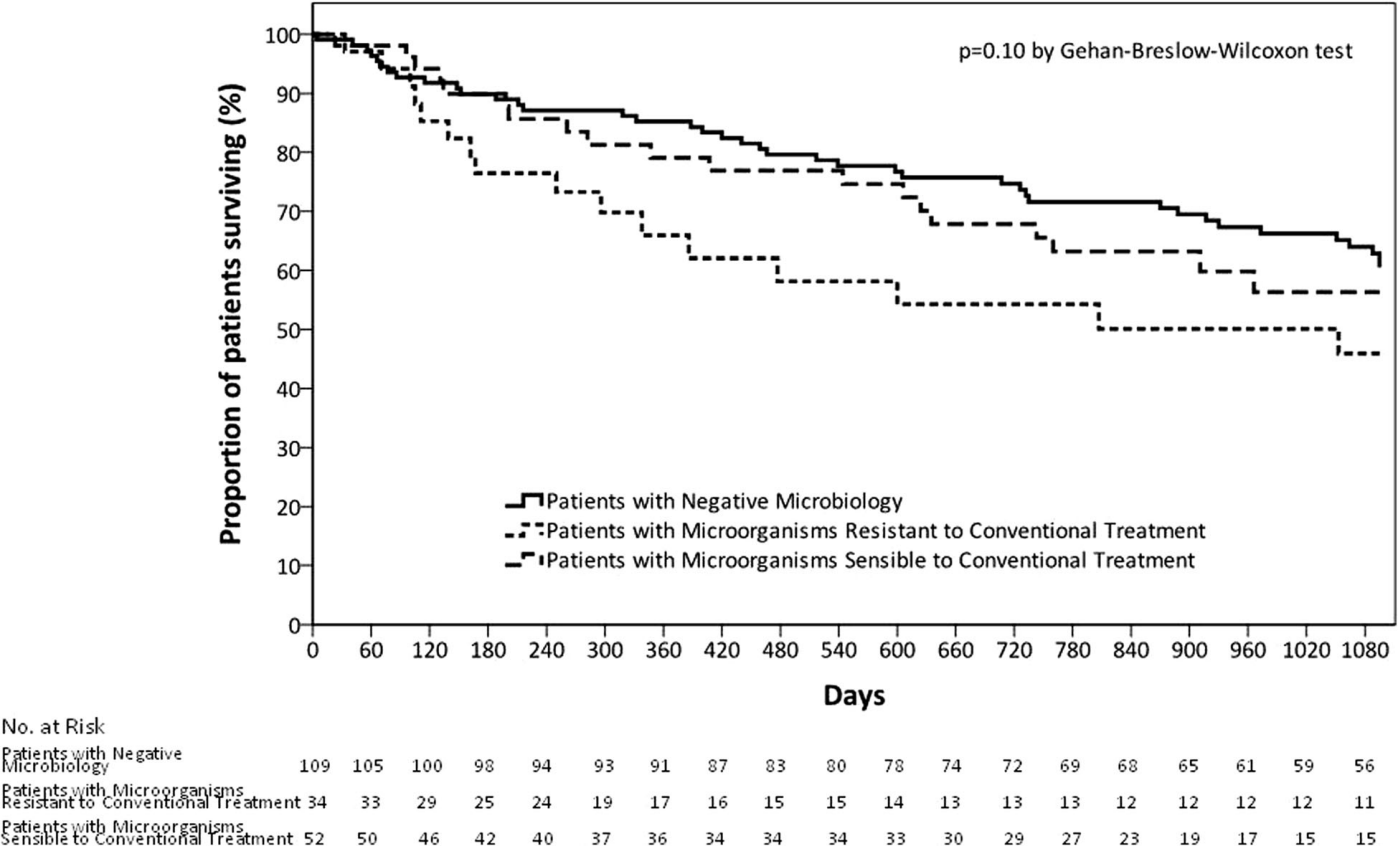
Kaplan–Meier analysis of the effect of microbial aetiology groups on time to death

Patients with MRCT had longer median hospital stays than the other 2 groups (9 days [7–14] vs. 8 days [6–10] in both cases, respectively; *p* = 0.026) (Table 3). No differences were observed in ICU admission, IMV or NIMV rates among the groups. Mortality rates were comparable at 30-days, one year and 3 years, and the number of AECOPD after discharge did not differ among the groups. The Kaplan-Meier survival curves depicting the 3-year mortality rates as a function of the 3 microbial aetiology groups are shown in Fig. 4. No differences were observed when comparing patients with adequate and inadequate empiric antibiotic treatment in each group (MRCT and MSCT) or in overall population (Additional file 1: Table S5).

The simple linear regression analysis revealed several variables significantly associated with length of hospital stay (Table 4). BODeX index, CRP levels, requirement of mechanical ventilation (invasive or non-invasive) and isolation of MRCT were those independently associated with length of hospital stay in the multiple analyses. The data for internal validation of the linear regression model (using bootstrapping with 1000 samples) are presented in Additional file 1: Table S8. The variables included in the model demonstrated robust results, with small 95% CIs around the original coefficients.

**Table 4:** Significant simple and multiple linear regression analyses of associations of risk of length of hospital stay

Variable	Simple			Multiple^a^		
	*β*	95% CI	*P* value	*β*	95% CI	*P* value
≥ 2 AECOPD or 1 admission by AECOPD in the previous year	1.89	0.05 a 3.72	0.044	–	–	–
Bronchiectasis	1.70	−0.19 a 3.61	0.079	–	–	–
Long-term oxygen therapy	1.73	−0.19 a 3.66	0.077	–	–	–
BODEx index	0.89	0.08 a 1.70	0.032	0.81	0.04 a 1.59	0.039
C-reactive protein (mg/dL)	−1.56	−3.37 a 0.26	0.092	–	–	–
PaCO_2_	2.14	0.34 a 3.94	0.020	–	–	–
Previous positive sputum culture for *Pseudomonas aeruginosa*	5.88	1.83 a 9.92	0.005	–	–	–
Adequate Empiric Treatment	−1.54	−2.86 a − 0.22	0.023	–	–	–
Invasive mechanical ventilation	8.06	2.91 a 13.21	0.002	5.92	0.82 a 11.02	0.023
Non-invasive mechanical ventilation	3.82	1.45 a 6.19	0.002	3.18	0.84 a 5.53	0.008
MRCT Isolation	3.53	1.19 a 5.88	0.003	3.11	0.84 a 5.37	0.007

*Abbreviations: β* unstandardized beta coefficient, *AECOPD* indicates acute exacerbation of chronic obstructive pulmonary disease exacerbation, *BODEx* body mass index, airflow obstruction, dyspnoea and exacerbations, *CI* confidence interval

^a^Adjusted R^2^ coefficient of determination = 11.8%

Patients with *P. aeruginosa* isolation in the MRCT group had higher hospital stays than patients without *P. aeruginosa* in the same group. Patients with isolation of MDR or XDR *P. aeruginosa* had the longest stays, but there was no difference in mortality (Additional file 1: Table S3, Table S4, Figure S1). The 3-year mortality among patients with < 2 AECOPD episodes and no admissions for AECOPD in the previous year differed significantly between groups in the Kaplan-Meier analysis (*p* = 0.019; Additional file 1: Figure S2).

## Discussion

In our study, we analysed 3 well-characterised groups, comparing patients with MRCTs against controls groups of patients with MSCTs and patients with negative microbiology and no previous antibiotic use. Our analyses revealed that not currently smoking, ≥ 2 AECOPD episodes or ≥ 1 admission for AECOPD in the last year, and a low systemic inflammatory response at admission were independent risk factors for AECOPD caused by an MRCT. However, although patients with MRCT had longer hospital stays, they did not have higher mortality or more severe AECOPD than the control groups. At baseline, patients with MRCT had more severe disease, as measured by the dyspnoea scale, COPDSS scale, BODEx index and history of previous AECOPD. There were no differences in symptoms or pulmonary gas exchange features at admission.

AECOPD are events that mark disease progression, and as taken into account by the GOLD guidelines [5], are as important as airflow limitation. Indeed, it is evident that there are patients who are susceptible to frequent exacerbations, and in these, the most important predictor of future episodes is the history of AECOPD [22]; however, the association with microbiologic data has been poorly analysed to date.

A low systemic inflammatory response was a risk factor for MRCT isolation in this study, which could be due to lower virulence or reduced ability to produce acute phase reactants in the presence of these microorganisms. Similar results were observed in patients with community acquired pneumonia or ventilator associated pneumonia in whom *P. aeruginosa* was isolated [23, 24].

There was also an association between smoking status and MRCT isolation, specifically in favour of non-current smoking status. It is known that smoke increases upper respiratory tract colonisation of *S. pneumoniae, H. influenzae, M. catarrhalis* and *Streptococcus pyogenes* [25], and that smoking facilitates colonisation of the lung with these bacteria [9]. This is probably related to the decreased phagocytic ability of alveolar macrophages and the decreased cytokine response associated with smoking [26, 27]. The association between smoking status and MRCT isolation in this study should not, therefore, weaken the recommendation for smoking cessation for all patients. Other explanations to this point maybe those individuals who develop respiratory symptoms due to more severe disease being more likely to quit smoking.

Previous studies have produced controversial data about the presence of *P. aeruginosa* in isolates, though they have tended to show that sensitive *P. aeruginosa* had higher mortality [28–31]. We found no differences in mortality between patients with *P. aeruginosa*, including those with MDR strains. In other respiratory diseases, such as cystic fibrosis or non-cystic fibrosis bronchiectasis, microbiologic isolation of *P. aeruginosa* and MRSA has been shown to have an important role in disease progression [32–36]. Although there is evidence that eradication with antibiotic treatment would be beneficial in these diseases, there is no such evidence that similar benefits would exist for patients with COPD.

The role of antimicrobial treatment remains controversial in AECOPD. With the exception of patients who require mechanical ventilation and ICU admission, the benefits of antibiotic treatment are limited, and are mainly observed in patients with purulent sputum or in those with AECOPD graded as type I by the Anthonisen classification [37–41]. The effect of inadequate antibiotic treatment is poorly understood in patients with AECOPD. In this study, we did not observe any differences in outcomes between patients with inadequate and adequate empiric antibiotic treatment.

The predictive factors identified in this study represent the first step in the development of a prediction model. To move forward, the potential model will need to undergo external validation with larger patient cohorts from multiple centres. We could also apply the results of internal validation techniques to understand how likely this model will be replicable to future studies and to studies at other centres. Bootstrapping techniques were applied to our data, and the results indicated that the coefficients obtained from the prediction model were quite robust. Notably, previous *P. aeruginosa* isolation was the one factor that the bootstrap analysis indicated might have limited repeatability in future work. Thus, we opted to remove previous *P. aeruginosa* isolation from the overall model and include it in a specific multivariate analysis for *P. aeruginosa.* In the real-world clinical setting where this prediction model could be used, previous *P. aeruginosa* isolation remains an important clinical characteristic that can play a substantial role in decision making.

We did not observe differences in the majority of outcomes when comparing MRCT vs non-MRCT exacerbations. However; length of stay was longer in the MRCT group. This is an important outcome to be taken into account to make efforts in predicting and treating these microorganisms in AECOPD.

Our study has some limitations that should be acknowledged. First, the study was carried out at only one centre in Spain. Second, the small sample limited the analysis of specific factors per bacterium. There is limited information about MRCT isolation in patients with AECOPD, and where there is, it is mainly for bacteria other than *P. aeruginosa.* A confirmation of our results in a large and well balanced, international cohort of AECOPD is therefore desirable. Finally, other limitation of this study was the use of sputum cultures and the potential difficulty to distinguish between colonization and infection. However, we only accepted samples of good quality and we did not culture those of low quality. In addition this is the usual way to diagnose lower airway infection in AECOPD in the majority of studies, given that performing bronchoscopy in these patients is extremely difficult. Moreover we validated sputum cultures some years ago in comparison with bronchoscopic samples [42].

## Conclusions

In conclusion, non-current smoking status, ≥2 AECOPD episodes or ≥ 1 admissions for AECOPD in the previous year, and low systemic inflammatory response are independent risk factors to have an AECOPD caused by a MRCT. Length of stay was significantly longer in AECOPD caused by MRCT microorganisms.

## Additional file

Additional file 1: **Table S1.** Microbiological Isolations. **Table S2.** Internal Validation of the Multivariate Logistic Regression Model using Bootstrap Method. **Table S3.** Multinomial Logistic Regression Model for Microorganisms Resistant to Conventional Treatment (with *Pseudomonas aeruginosa*) or Microorganisms Sensitive to Conventional Treatment Relative to Negative Microbiology. **Table S4.** Internal Validation of the Multinomial Logistic Regression Model for Microorganisms Resistant to Conventional Treatment (with *Pseudomonas aeruginosa*) or Microorganisms Sensitive to Conventional Treatment using Bootstrap Method. **Table S5.** Outcomes according to Appropriateness of Empiric Treatment. **Table S6**. Comparison of Outcomes between Patients with *Pseudomonas Aeruginosa* and Patients without *Pseudomonas Aeruginosa* in Microorganisms Resistant to Conventional Treatment Group. **Table S7.** Comparison between *Pseudomonas Aeruginosa* MDR/XDR Isolation with other Microorganism Isolated in Microorganisms Resistant to Conventional Treatment Group. **Table S8.** Internal Validation of Risk of Length of Hospital Stay Using Bootstrap Technique. **Figure S1.** Receiver Operating Characteristic Curve for Multinomial Logistic Regression Model to *Pseudomonas aeruginosa.* **Figure S2.** Kaplan–Meier Analysis of the Effect of Microbial Aetiology Groups on Time to Death. **Figure S3.** Kaplan–Meier Analysis of the Effect of Microbial Aetiology Groups on Time to Death. A) Patients with < 2 AECOPD and none admission by AECOPD in the previous year; B) Patients with ≥ 2 AECOPD or 1 admission by AECOPD in the previous year. (DOC 265 kb)

## Abbreviations

AECOPD: Acute exacerbations of chronic obstructive pulmonary disease
BMI: Body mass index
CI: Confidence intervals
COPD: Chronic obstructive pulmonary disease
COPDSS: COPD severity score
GOLD: Global Initiative for Chronic Obstructive Lung Disease
LTOT: Long-term oxygen therapy
MDR: Multidrug-resistant
mMRC: Modified medical research council
MRCT: Microorganism resistant to conventional treatment
MRSA: Methicillin-resistant *Staphylococcus aureus*
MSCT: Microorganisms sensitive to conventional antimicrobial treatment
OR: Odds ratios
ROC: Receiver operating characteristic
XDR: Extensively drug resistant

## References

[1] Soler-Cataluña JJ, Martínez-García MA, Román Sánchez P, Salcedo E, Navarro M, Ochando R (2005). Severe acute exacerbations and mortality in patients with chronic obstructive pulmonary disease. Thorax.

[2] Soler N, Torres A, Ewig S, Gonzalez J, Celis R, El-Ebiary M (1998). Bronchial microbial patterns in severe exacerbations of chronic obstructive pulmonary disease (COPD) requiring mechanical ventilation. Am J Respir Crit Care Med.

[3] Wedzicha JA, Seemungal TAR (2007). COPD exacerbations: defining their cause and prevention. Lancet Lond Engl.

[4] White AJ, Gompertz S, Stockley RA (2003). Chronic obstructive pulmonary disease. 6: the aetiology of exacerbations of chronic obstructive pulmonary disease. Thorax.

[5] From the Global Strategy for the Diagnosis, Management and Prevention of COPD Global Initiative for Chronic Obstructive Lung Disease (GOLD) 2017. Available http://www.goldcopd.org/. Accessed 1 Nov 2018.

[6] Woodhead M, Blasi F, Ewig S, Huchon G, Leven M, Ortqvist A (2005). Guidelines for the management of adult lower respiratory tract infections. Eur Respir J.

[7] Miravitlles M, Espinosa C, Fernández-Laso E, Martos JA, Maldonado JA, Gallego M (1999). Relationship between bacterial flora in sputum and functional impairment in patients with acute exacerbations of COPD. Study Group of Bacterial Infection in COPD. Chest.

[8] Eller J, Ede A, Schaberg T, Niederman MS, Mauch H, Lode H (1998). Infective exacerbations of chronic bronchitis: relation between bacteriologic etiology and lung function. Chest.

[9] Soler N, Ewig S, Torres A, Filella X, Gonzalez J, Zaubet A (1999). Airway inflammation and bronchial microbial patterns in patients with stable chronic obstructive pulmonary disease. Eur Respir J.

[10] Rosell A, Monsó E, Soler N, Torres F, Angrill J, Riise G (2005). Microbiologic determinants of exacerbation in chronic obstructive pulmonary disease. Arch Intern Med.

[11] Celli BR, Barnes PJ (2007). Exacerbations of chronic obstructive pulmonary disease. Eur Respir J.

[12] Leclercq R, Cantón R, Brown DFJ, Giske CG, Heisig P, MacGowan AP (2013). EUCAST expert rules in antimicrobial susceptibility testing. Clin Microbiol Infect Off Publ Eur Soc Clin Microbiol Infect Dis.

[13] Magiorakos A-P, Srinivasan A, Carey RB, Carmeli Y, Falagas ME, Giske CG (2012). Multidrug-resistant, extensively drug-resistant and pandrug-resistant bacteria: an international expert proposal for interim standard definitions for acquired resistance. Clin Microbiol Infect Off Publ Eur Soc Clin Microbiol Infect Dis..

[14] Murray PR, Washington JA (1975). Microscopic and baceriologic analysis of expectorated sputum. Mayo Clin Proc.

[15] Charlson ME, Pompei P, Ales KL, MacKenzie CR (1987). A new method of classifying prognostic comorbidity in longitudinal studies: development and validation. J Chronic Dis.

[16] Miravitlles M, Llor C, de Castellar R, Izquierdo I, Baró E, Donado E (2009). Validation of the COPD severity score for use in primary care: the NEREA study. Eur Respir J.

[17] Soler-Cataluña JJ, Martínez-García MA, Sánchez LS, Tordera MP, Sánchez PR (2009). Severe exacerbations and BODE index: two independent risk factors for death in male COPD patients. Respir Med.

[18] Healey JF. Statistics: a tool for social research. 9th ed: Wadsworth: Cengage Learning; 2011.

[19] Efron B, Tibshirani RJ. An introduction to the bootstrap: CRC Press; 1994.

[20] Sterne JAC, White IR, Carlin JB, Spratt M, Royston P, Kenward MG (2009). Multiple imputation for missing data in epidemiological and clinical research: potential and pitfalls. BMJ.

[21] Steyerberg E (2009). Clinical prediction models: a practical Aapproach to development, validation, and updating.

[22] Hurst JR, Vestbo J, Anzueto A, Locantore N, Müllerova H, Tal-Singer R (2010). Susceptibility to exacerbation in chronic obstructive pulmonary disease. N Engl J Med.

[23] Cillóniz C, Gabarrús A, Ferrer M, Puig de la Bellacasa J, Rinaudo M, Mensa J, et al. Community-acquired pneumonia due to multidrug and non-multidrug resistant Pseudomonas aeruginosa. Chest 2016. 10.1016/j.chest.2016.03.042 27060725

[24] Fernández-Barat L, Ferrer M, De Rosa F, Gabarrús A, Esperatti M, Terraneo S (2017). Intensive care unit-acquired pneumonia due to Pseudomonas aeruginosa with and without multidrug resistance. J Inf Secur.

[25] Brook I, Gober AE (2005). Recovery of potential pathogens and interfering bacteria in the nasopharynx of smokers and nonsmokers. Chest.

[26] Berenson CS, Garlipp MA, Grove LJ, Maloney J, Sethi S (2006). Impaired phagocytosis of nontypeable Haemophilus influenzae by human alveolar macrophages in chronic obstructive pulmonary disease. J Infect Dis.

[27] Berenson CS, Wrona CT, Grove LJ, Maloney J, Garlipp MA, Wallace PK (2006). Impaired alveolar macrophage response to Haemophilus antigens in chronic obstructive lung disease. Am J Respir Crit Care Med.

[28] Renom F, Yáñez A, Garau M, Rubí M, Centeno M-J, Gorriz M-T (2010). Prognosis of COPD patients requiring frequent hospitalization: role of airway infection. Respir Med.

[29] Boutou AK, Raste Y, Reid J, Alshafi K, Polkey MI, Hopkinson NS (2014). Does a single Pseudomonas aeruginosa isolation predict COPD mortality?. Eur Respir J.

[30] Garcia-Vidal C, Almagro P, Romaní V, Rodríguez-Carballeira M, Cuchi E, Canales L (2009). Pseudomonas aeruginosa in patients hospitalised for COPD exacerbation: a prospective study. Eur Respir J.

[31] Rodrigo-Troyano A, Suarez-Cuartin G, Peiró M, Barril S, Castillo D, Sanchez-Reus F (2016). Pseudomonas aeruginosa resistance patterns and clinical outcomes in hospitalized exacerbations of COPD. Respirol Carlton Vic.

[32] Finch S, McDonnell MJ, Abo-Leyah H, Aliberti S, Chalmers JD (2015). A comprehensive analysis of the impact of Pseudomonas aeruginosa colonization on prognosis in adult bronchiectasis. Ann Am Thorac Soc.

[33] Wilson R, Aksamit T, Aliberti S, De Soyza A, Elborn JS, Goeminne P (2016). Challenges in managing Pseudomonas aeruginosa in non-cystic fibrosis bronchiectasis. Respir Med.

[34] Wilson CB, Jones PW, O’Leary CJ, Hansell DM, Cole PJ, Wilson R (1997). Effect of sputum bacteriology on the quality of life of patients with bronchiectasis. Eur Respir J.

[35] Sagel SD, Gibson RL, Emerson J, McNamara S, Burns JL, Wagener JS (2009). Impact of pseudomonas and staphylococcus infection on inflammation and clinical status in young children with cystic fibrosis. J Pediatr.

[36] Emerson J, Rosenfeld M, McNamara S, Ramsey B, Gibson RL (2002). Pseudomonas aeruginosa and other predictors of mortality and morbidity in young children with cystic fibrosis. Pediatr Pulmonol.

[37] Vollenweider DJ, Jarrett H, Steurer-Stey CA, Garcia-Aymerich J, Puhan MA (2012). Antibiotics for exacerbations of chronic obstructive pulmonary disease. Cochrane Database Syst Rev.

[38] Nouira S, Marghli S, Belghith M, Besbes L, Elatrous S, Abroug F (2001). Once daily oral ofloxacin in chronic obstructive pulmonary disease exacerbation requiring mechanical ventilation: a randomised placebo-controlled trial. Lancet Lond Engl..

[39] Saint S, Bent S, Vittinghoff E, Grady D (1995). Antibiotics in chronic obstructive pulmonary disease exacerbations. A meta-analysis. JAMA.

[40] Wilson R, Jones P, Schaberg T, Arvis P, Duprat-Lomon I, Sagnier PP (2006). Antibiotic treatment and factors influencing short and long term outcomes of acute exacerbations of chronic bronchitis. Thorax.

[41] Soler N, Esperatti M, Ewig S, Huerta A, Agustí C, Torres A (2012). Sputum purulence-guided antibiotic use in hospitalised patients with exacerbations of COPD. Eur Respir J.

[42] Soler N, Agustí C, Angrill J, Puig De la Bellacasa J, Torres A (2007). Bronchoscopic validation of the significance of sputum purulence in severe exacerbations of chronic obstructive pulmonary disease. Thorax.

