# Supplementary material for: Microorganisms resistant to conventional antimicrobials in acute exacerbations of chronic obstructive pulmonary disease
Source: Respir Res. 2018 Jun 15;19:119. doi: 10.1186/s12931-018-0820-1 (PMC6003174; doi:10.1186/s12931-018-0820-1)
Supplement: Supplementary file 1 — Table S1. Microbiological Isolations. Table S2. Internal Validation of the Multivariate Logistic Regression Model using Bootstrap Method. Table S3. Multinomial Logistic Regression Model for Microorganisms Resistant to Conventional Treatment (with Pseudomonas aeruginosa) or Microorganisms Sensitive to Conventional Treatment Relative to Negative Microbiology. Table S4. Internal Validation of the Multinomial Logistic Regression Model for Microorganisms Resistant to Conventional Treatment (with Pseudomonas aeruginosa) or Microorganisms Sensitive to Conventional Treatment using Bootstrap Method. Table S5. Outcomes according to Appropriateness of Empiric Treatment. Table S6. Comparison of Outcomes between Patients with Pseudomonas Aeruginosa and Patients without Pseudomonas Aeruginosa in Microorganisms Resistant to Conventional Treatment Group. Table S7. Comparison between Pseudomonas Aeruginosa MDR/XDR Isolation with other Microorganism Isolated in Microorganisms Resistant to Conventional Treatment Group. Table S8. Internal Validation of Risk of Length of Hospital Stay Using Bootstrap Technique. Figure S1. Receiver Operating Characteristic Curve for Multinomial Logistic Regression Model to Pseudomonas aeruginosa. Figure S2. Kaplan–Meier Analysis of the Effect of Microbial Aetiology Groups on Time to Death. Figure S3. Kaplan–Meier Analysis of the Effect of Microbial Aetiology Groups on Time to Death. A) Patients with < 2 AECOPD and none admission by AECOPD in the previous year; B) Patients with ≥ 2 AECOPD or 1 admission by AECOPD in the previous year. (DOC 265 kb) [file 12931_2018_820_MOESM1_ESM.doc]

**Additional file**

**Microorganisms Resistant to Conventional Antimicrobials in Acute Exacerbations of Chronic Obstructive Pulmonary Disease**

Cristina Estirado, MD^1*^; Adrian Ceccato, MD^2*^; Monica Guerrero, MD, PhD^2^, Arturo Huerta, MD, PhD^2^; Catia Cilloniz, PhD^2^; Olivia Vilaró, RN^2^; Albert Gabarrús, MSc^2^; Joaquim Gea, MD, PhD^1^; Ernesto Crisafulli, MD, PhD^3^; Nestor Soler, MD, PhD^2^; Antoni Torres, MD, PhD^2^

**Table S1. Microbiological Isolations**

|  | **Patients with Microorganisms Resistant to Conventional Treatment**  **(n = 34)** | **Patients with Microorganisms Sensitive to Conventional Treatment**  **(n = 52)** |
| --- | --- | --- |
| *Pseudomonas aeruginosa*, n (%) | 25 (74) | 0 |
| *Streptococcus pneumoniae*, n (%) | 0 | 18 (35) |
| *Haemophilus influenzae*, n (%) | 0 | 16 (31) |
| *Staphylococcus aureus*, n (%) | 2 (6) | 5 (10) |
| *Moraxella catarrhalis*, n (%) | 0 | 3 (6) |
| *Stenotrophomonas maltophilia*, n (%) | 1 (3) | 0 |
| *Serratia* spp., n (%) | 0 | 1 (2) |
| *Klebsiella* spp., n (%) | 0 | 1 (2) |
| *Acinetobacter* *baumannii*, n (%) | 1 (3) | 0 |
| Polymicrobial, n (%)^a^ | 5 (15) | 2 (4) |
| Others, n (%) | 0 | 6 (12) |

^a^ Polymicrobial Isolation includes: *3 Pseudomonas aeruginosa, 2 Stenotrophomonas maltophilia, 1 Staphylococcus aureus, 3 Streptococcus pneumoniae, 1 others.*

**Table S2. Internal Validation of the Multivariate Logistic Regression Model using Bootstrap Method**

| **Group** | **Variable** | **Original** | **Bias** | **Standard Error** | ***P* value** | **95% Confidence Interval** | |
| --- | --- | --- | --- | --- | --- | --- | --- |
|  |  |  |  |  |  | **Lower** | **Upper** |
| Patients with Microorganisms Resistant to Conventional Treatment | Intercept | -3.982 | -0.681 | 2.669 | 0.021 | -7.271 | -2.876 |
|  | Non-current smoker | 1.433 | 0.515 | 2.589 | 0.016 | 0.360 | 3.676 |
|  | ≥2 AECOPD or 1 admission by AECOPD in the previous year | 1.417 | 0.086 | 0.503 | 0.001 | 0.550 | 2.493 |
|  | BODEx index 4th quartile: 7-9 | -0.727 | -0.966 | 4.288 | 0.363 | -20.469 | 0.801 |
|  | BODEx index 3rd quartile: 5-6 | 0.613 | 0.058 | 0.632 | 0.282 | -0.537 | 1.983 |
|  | BODEx index 2nd quartile: 3-4 | 0.840 | 0.018 | 0.724 | 0.182 | -0.504 | 2.253 |
|  | C-reactive protein <5 mg/dL at admission | 1.274 | 0.094 | 0.508 | 0.002 | 0.447 | 2.501 |
| Patients with Microorganisms Sensitive to Conventional Treatment | Intercept | -0.631 | -0.012 | 0.424 | 0.102 | -1.534 | 0.191 |
|  | Non-current smoker | -0.250 | -0.002 | 0.401 | 0.512 | -0.981 | 0.513 |
|  | ≥2 AECOPD or 1 admission by AECOPD in the previous year | 0.558 | 0.023 | 0.490 | 0.234 | -0.370 | 1.530 |
|  | BODEx index 4th quartile: 7-9 | -1.935 | -2.655 | 6.655 | 0.006 | -22.123 | -0.723 |
|  | BODEx index 3rd quartile: 5-6 | 0.112 | 0.001 | 0.543 | 0.819 | -1.039 | 1.170 |
|  | BODEx index 2nd quartile: 3-4 | -0.473 | -0.136 | 0.981 | 0.419 | -2.262 | 0.683 |
|  | C-reactive protein <5 mg/dL at admission | 0.129 | 0.002 | 0.378 | 0.738 | -0.639 | 0.857 |

*Abbreviations:* AECOPD indicates acute exacerbation of chronic obstructive pulmonary disease exacerbation; BODEx, body mass index, airflow obstruction, dyspnoea and exacerbations.

**Table S3. Multinomial Logistic Regression Model for Microorganisms Resistant to Conventional Treatment (with *Pseudomonas aeruginosa*) or Microorganisms Sensitive to Conventional Treatment Relative to Negative Microbiology**

| **Variable** | **Patients with Microorganisms Resistant to Conventional Treatment (with *Pseudomonas aeruginosa*)** | | | **Patients with Microorganisms Sensitive to Conventional Treatment** | | |
| --- | --- | --- | --- | --- | --- | --- |
|  | **OR** | **95% CI** | ***P* value** | **OR** | **95% CI** | ***P* value** |
| Non-current smoker | 2.38 | 0.69 to 8.16 | 0.17 | 0.77 | 0.38 to 1.58 | 0.47 |
| ≥2 AECOPD or 1 admission by AECOPD in the previous year | 3.08 | 0.98 to 9.64 | **0.054** | 1.75 | 0.76 to 4.02 | 0.19 |
| BODEx index |  |  |  |  |  |  |
| 1st quartile: 0-2 | 1 | - | - | 1 | - | - |
| 2nd quartile: 3-4 | 2.64 | 0.61 to 11.54 | 0.20 | 0.62 | 0.21 to 1.89 | 0.41 |
| 3rd quartile: 5-6 | 2.52 | 0.65 to 9.68 | 0.18 | 1.10 | 0.42 to 2.84 | 0.85 |
| 4th quartile: 7-9 | 0.69 | 0.12 to 3.91 | 0.67 | 0.14 | 0.03 to 0.69 | **0.016** |
| C-reactive protein <5 mg/dL at admission | 4.18 | 1.36 to 12.87 | **0.013** | 1.17 | 0.59 to 2.34 | 0.65 |
| Previous positive sputum culture for *Pseudomonas aeruginosa* | 38.61 | 3.59 to 415.66 | **0.003** | 2.33 | 0.13 to 40.55 | 0.54 |

*Abbreviations:* AECOPD indicates acute exacerbation of chronic obstructive pulmonary disease exacerbation; BODEx, body mass index, airflow obstruction, dyspnoea and exacerbations; CI, confidence interval; OR: odds ratio. Data are shown as estimated ORs (95% CIs) of the explanatory variables observed at admission of patients in the microorganisms resistant to conventional treatment (with *Pseudomonas aeruginosa*) (MRCT with *Pseudomonas aeruginosa*) and microorganisms sensitive to conventional treatment (MSCT) groups. The OR is defined as the probability of membership of the groups MRCT with *Pseudomonas aeruginosa* or MSCT divided by the probability of membership of the negative microbiology group. The P value is based on the null hypothesis that all ORs relating to an explanatory variable equal unity. Model characteristics: likelihood ratio X^2^ test, p = 0.88; R^2^ coefficients = 0.31 (Cox and Snell), 0.36 (Nagelkerke).

**Table S4. Internal Validation of the Multinomial Logistic Regression Model for Microorganisms Resistant to Conventional Treatment (with *Pseudomonas aeruginosa*) or Microorganisms Sensitive to Conventional Treatment using Bootstrap Method**

| **Group** | **Variable** | **Original** | **Bias** | **Standard Error** | ***P* value** | **95% Confidence Interval** | |
| --- | --- | --- | --- | --- | --- | --- | --- |
|  |  |  |  |  |  | **Lower** | **Upper** |
| Patients with Microorganisms Resistant to Conventional Treatment (with *Pseudomonas aeruginosa*) | Intercept | -4.114 | -0.854 | 2.702 | 0.001 | -17.152 | -2.871 |
|  | Non-current smoker | 0.868 | 0.540 | 2.444 | 0.154 | -0.315 | 12.321 |
|  | ≥2 AECOPD or 1 admission by AECOPD in the previous year | 1.123 | 0.126 | 0.587 | 0.025 | 0.180 | 2.505 |
|  | BODEx index 4th quartile: 7-9 | -0.376 | -1.047 | 3.679 | 0.608 | -15.481 | 1.228 |
|  | BODEx index 3rd quartile: 5-6 | 0.923 | 0.009 | 0.724 | 0.134 | -0.526 | 2.559 |
|  | BODEx index 2nd quartile: 3-4 | 0.972 | -0.043 | 1.539 | 0.235 | -1.040 | 2.851 |
|  | C-reactive protein <5 mg/dL at admission | 1.430 | 0.206 | 1.009 | 0.008 | 0.378 | 3.141 |
|  | Previous positive sputum culture for *Pseudomonas aeruginosa* | 3.653 | 6.448 | 9.673 | 0.016 | 1.183 | 23.902 |
| Patients with Microorganisms Sensitive to Conventional Treatment | Intercept | -0.644 | -0.023 | 0.412 | 0.101 | -1.504 | 0.123 |
|  | Non-current smoker | -0.263 | 0.013 | 0.400 | 0.500 | -1.017 | 0.511 |
|  | ≥2 AECOPD or 1 admission by AECOPD in the previous year | 0.559 | 0.012 | 0.489 | 0.222 | -0.459 | 1.489 |
|  | BODEx index 4th quartile: 7-9 | -1.955 | -2.172 | 5.349 | 0.006 | -18.195 | -0.729 |
|  | BODEx index 3rd quartile: 5-6 | 0.091 | 0.008 | 0.538 | 0.872 | -1.021 | 1.108 |
|  | BODEx index 2nd quartile: 3-4 | -0.470 | -0.125 | 0.998 | 0.427 | -1.961 | 0.674 |
|  | C-reactive protein <5 mg/dL at admission | 0.159 | -0.012 | 0.377 | 0.652 | -0.586 | 0.902 |
|  | Previous positive sputum culture for *Pseudomonas aeruginosa* | 0.848 | -0.308 | 12.735 | 0.167 | -18.482 | 19.531 |

*Abbreviations:* AECOPD indicates acute exacerbation of chronic obstructive pulmonary disease exacerbation; BODEx, body mass index, airflow obstruction, dyspnoea and exacerbations.

**Table S5. Outcomes according to Appropriateness of Empiric Treatment**

| **Variable** | **Inadequate Empiric Treatment** | | | | **Adequate Empiric Treatment** | | | |  |  |  |
| --- | --- | --- | --- | --- | --- | --- | --- | --- | --- | --- | --- |
|  | **Patients with Microorganisms Resistant to Conventional Treatment**  **(n = 15)** | **Patients with Microorganisms Sensible to Conventional Treatment**  **(n = 5)** | **Total**  **(n = 20)** | ***P* value** | **Patients with Microorganisms Resistant to Conventional Treatment**  **(n = 19)** | **Patients with Microorganisms Sensible to Conventional Treatment**  **(n = 47)** | **Total**  **(n = 66)** | ***P* value** | ***P* value^a^** | ***P* value^b^** | ***P* value^c^** |
| AECOPD after 30 days of discharge, n (%) | 10 (72) | 1 (25) | 11 (61) | 0.25 | 14 (74) | 19 (50) | 33 (58) | 0.088 | >0.99 | 0.61 | 0.81 |
| Number of AECOPD after 30 days of discharge, median (IQR) | 1 (0; 3) | 0 (0; 0.5) | 1 (0; 2) | 0.076 | 1 (0; 3) | 0.5 (0; 2) | 1 (0; 2) | 0.22 | 0.94 | 0.23 | 0.94 |
| Time to the next AECOPD, median (IQR), days | 35 (25; 170) | 126 (126; 126) | 55 (30; 167) | 0.66 | 40 (19; 144) | 51 (27; 166) | 48 (19; 166) | 0.75 | 0.98 | 0.69 | 0.99 |
| Length of stay, median (IQR), days | 9 (8; 14) | 10 (7; 17) | 9.5 (7.5; 15) | 0.90 | 9 (7; 19) | 8 (6; 10.5) | 8 (6; 12) | 0.11 | 0.99 | 0.39 | 0.19 |
| ICU admission, n (%) | 1(7) | 0 (0) | 1 (5) | >0.99 | 3 (16) | 6(15) | 9(15) | >0.99 | 0.61 | >0.99 | 0.44 |
| IMV, n (%) | 1(7) | 0 (0) | 1 (5) | >0.99 | 1(5) | 1(3) | 2 (3) | 0.52 | >0.99 | >0.99 | 0.57 |
| NIMV, n (%) | 3 (20) | 0 (0) | 3 (15) | 0.54 | 3 (16) | 10 (23) | 13 (21) | 0.74 | >0.99 | 0.57 | 0.75 |
| 30-day mortality, n (%) | 1(7) | 0 (0) | 1 (5) | >0.99 | 0 (0) | 1(3) | 1 (2) | >0.99 | 0.44 | >0.99 | 0.43 |
| 1-year mortality, n (%) | 6 (40) | 1 (20) | 7 (35%) | 0.61 | 5 (26) | 11 (25) | 16 (25) | >0.99 | 0.48 | >0.99 | 0.40 |
| 3-years mortality, n (%) | 9 (69) | 1 (50) | 10 (67) | >0.99 | 7 (50) | 18 (62) | 25 (58) | 0.52 | 0.31 | >0.99 | 0.56 |

Abbreviations: AECOPD indicates acute exacerbation of chronic obstructive pulmonary disease exacerbation; ICU, intensive care unit; IMV, invasive mechanical ventilation; IQR, interquartile range; NIMV, non-invasive mechanical ventilation. Data are shown as number of patients (%), or median (1^st^ quartile; 3^rd^ quartile). Percentages are calculated on non-missing data. ^a^ P values are for the comparison of inadequate empiric treatment/MRCT with adequate empiric treatment/MRCT. ^b^ P values are for the comparison of inadequate empiric treatment/MSCT with adequate empiric treatment/MSCT. ^c^ P values are for the comparison of inadequate empiric treatment/total with adequate empiric treatment/total.

**Table S6. Comparison of Outcomes between Patients with *Pseudomonas Aeruginosa* and Patients without *Pseudomonas Aeruginosa* in Microorganisms Resistant to Conventional Treatment Group**

| **Variable** | **Patients with *Pseudomonas aeruginosa* (n = 28)** | **Patients without *Pseudomonas aeruginosa* in patients with MRCT (n = 6)** | ***P* value** |
| --- | --- | --- | --- |
| AECOPD after 30 days of discharge, n (%) | 18 (67) | 6 (100) | 0.16 |
| Number of AECOPD after 30 days of discharge, median (IQR) | 1 (0; 2) | 2.5 (1; 3) | 0.092 |
| Time to the next AECOPD, median (IQR), days | 35 (19; 170) | 58 (35; 79) | 0.88 |
| Length of stay, median (IQR), days | 11 (8; 17.5) | 5 (4; 7) | 0.002 |
| ICU admission, n (%) | 4 (14) | 0 (0) | >0.99 |
| IMV, n (%) | 2 (7) | 0 (0) | >0.99 |
| NIMV, n (%) | 6 (21) | 0 (0) | 0.56 |
| 30-day mortality, n (%) | 1 (4) | 0 (0) | >0.99 |
| 1-year mortality, n (%) | 9 (32) | 2 (33) | >0.99 |
| 3-years mortality, n (%) | 14 (56) | 2 (100) | >0.99 |

Abbreviations: AECOPD indicates acute exacerbation of chronic obstructive pulmonary disease exacerbation; ICU, intensive care unit; IMV, invasive mechanical ventilation; IQR, interquartile range; NIMV, non-invasive mechanical ventilation. Data are shown as number of patients (%), or median (1^st^ quartile; 3^rd^ quartile). Percentages are calculated on non-missing data.

**Table S7. Comparison between *Pseudomonas Aeruginosa* MDR/XDR Isolation with other Microorganism Isolated in Microorganisms Resistant to Conventional Treatment Group**

| **Variable** | **Patients with *Pseudomonas aeruginosa* MDR and XDR (n = 13)** | **Patients without *Pseudomonas aeruginosa* MDR and XDR (n = 21)** | ***P* value** |
| --- | --- | --- | --- |
| AECOPD after 30 days of discharge, n (%) | 9 (75) | 15 (71) | >0.999 |
| Number of AECOPD after 30 days of discharge, median (IQR) | 1 (0.5; 3) | 1 (0; 2) | 0.892 |
| Time to the next AECOPD, median (IQR), days | 37 (19; 173) | 40 (25; 115) | 0.900 |
| Length of stay, median (IQR), days | 14 (10; 21) | 8 (7; 10) | **0.002** |
| ICU admission, n (%) | 3 (23) | 1 (5) | 0.274 |
| IMV, n (%) | 1 (8) | 1 (5) | >0.999 |
| NIMV, n (%) | 4 (31) | 2 (10) | 0.173 |
| 30-day mortality, n (%) | 1 (8) | 0 | 0.382 |
| 1-year mortality, n (%) | 5 (39) | 6 (29) | 0.709 |
| 3-years mortality, n (%) | 6 (55) | 10 (63) | 0.710 |

Abbreviations: AECOPD indicates acute exacerbation of chronic obstructive pulmonary disease exacerbation; ICU, intensive care unit; IMV, invasive mechanical ventilation; IQR, interquartile range; MDR, multi-drug resistant; NIMV, non-invasive mechanical ventilation; XDR extensive-drug resistant.

**Table S8. Internal Validation of Risk of Length of Hospital Stay Using Bootstrap Technique**

|  | **Original** | **Bias** | **SE** | ***p*-value** | **95% BCTo CI** | |
| --- | --- | --- | --- | --- | --- | --- |
| Constant | **6,628** | **,004** | **,667** | **,001** | **5,252** | **7,989** |
| BODEx index | **,814** | **-,009** | **,335** | **,027** | **,223** | **1,463** |
| Invasive mechanical ventilation | **5,919** | **,564** | **8,050** | **,492** | **-5,496** | **25,300** |
| Non-invasive mechanical ventilation | **3,183** | **,073** | **1,725** | **,061** | **-,049** | **6,774** |
| MRCT Isolation | **3,107** | **-,001** | **1,466** | **,039** | **,304** | **5,857** |

BCa indicates adjusted bootstrap; CI, confidence interval; SE, standard error

**Figure S1. Receiver Operating Characteristic Curve for Multinomial Logistic Regression Model to *Pseudomonas aeruginosa***

Abbreviations: AUC indicates area under the curve; CI, confidence interval.

**Figure S2. Kaplan–Meier Analysis of the Effect of Microbial Aetiology Groups on Time to Death**

**Figure S3. Kaplan–Meier Analysis of the Effect of Microbial Aetiology Groups on Time to Death. A) Patients with <2 AECOPD and none admission by AECOPD in the previous year; B) Patients with ≥2 AECOPD or 1 admission by AECOPD in the previous year.**

**A**

**
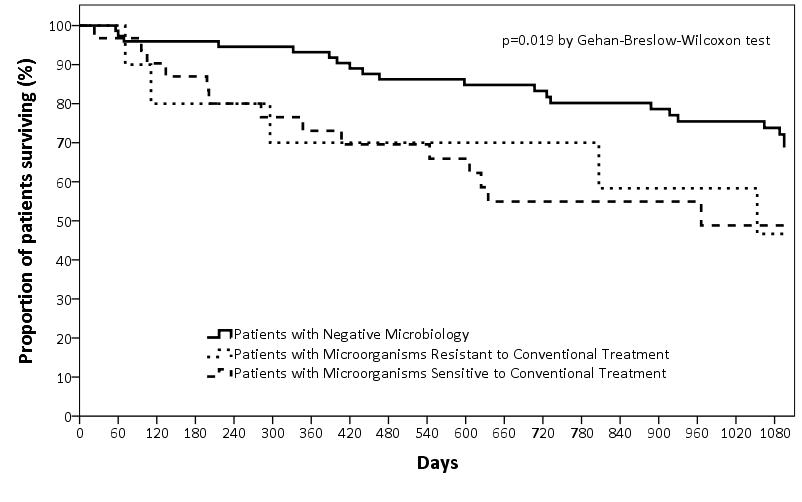
**

**B**

**
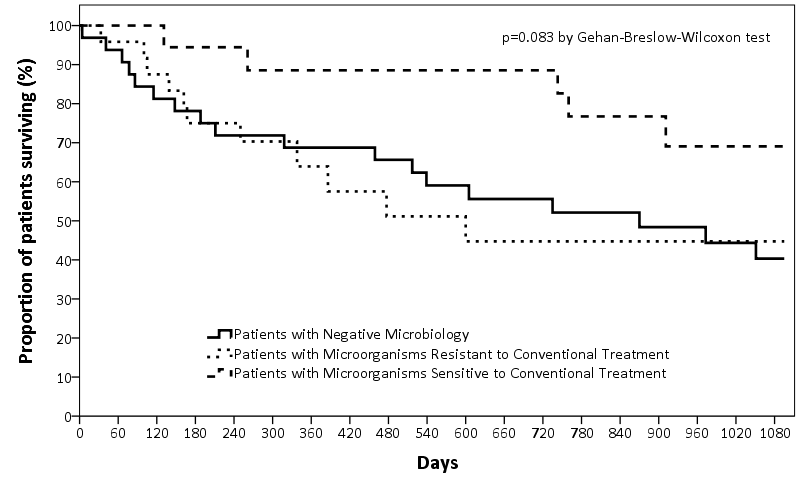
**

*Abbreviations:* AECOPD indicates acute exacerbation of chronic obstructive pulmonary disease exacerbation.
